# Fabrication and In-Vivo Study of Micro-Colloidal *Zanthoxylum acanthopodium*-Loaded Bacterial Cellulose as a Burn Wound Dressing

**DOI:** 10.3390/polym12071436

**Published:** 2020-06-27

**Authors:** Khatarina Meldawati Pasaribu, Saharman Gea, Syafruddin Ilyas, Tamrin Tamrin, Appealwan Altruistis Sarumaha, Ardiansyah Sembiring, Izabela Radecka

**Affiliations:** 1 Postgraduate School, Department of Chemistry, Faculty of Mathematics and Natural Sciences, Universitas Sumatera Utara, Jl. Bioteknologi No. 1, Medan 20155, Indonesia; khatarinameldawati@yahoo.co.id; 2 Department of Chemistry, Faculty of Mathematics and Natural Sciences, Universitas Sumatera Utara, Jl. Bioteknologi No. 1, Medan 20155, Indonesia; tamrin@usu.ac.id (T.T.); alwansarumaha17@gmail.com (A.A.S.); ardiansyahsembiring31@gmail.com (A.S.); 3 Department of Biology, Faculty of Mathematics and Natural Sciences, Universitas Sumatera Utara, Jl. Bioteknologi No. 1, Medan 20155, Indonesia; syafruddin6@usu.ac.id; 4 Wolverhampton School of Sciences, Faculty of Science and Engineering, University of Wolverhampton, Wulfruna Street, Wolverhampton WV1 1LY, UK; i.radecka@wlv.ac.uk

**Keywords:** bacterial cellulose, MZA, BC-MZA, composite, wound dressing

## Abstract

Bacterial cellulose (BC) is a biopolymer commonly used for wound dressing due to its high biocompatible properties either in-vitro or in-vivo. The three-dimensional fiber structure of BC becomes an advantage because it provides a template for the impregnation of materials in order to improve BC’s properties as a wound dressing, since BC has not displayed any bioactivity properties. In this study, micro-colloidal *Zanthoxylum acanthopodium* (MZA) fruit was loaded into BC fibers via an in-situ method. *Z. acanthopodium* is known to have anti-inflammatory, antioxidant and antimicrobial activities that can support BC to accelerate the wound healing process. The FTIR, XRD and SEM analysis results showed that the loading process of MZA and the composite fabrication were successfully carried out. The TGA test also showed that the presence of MZA in BC fibers decreased T_max_ composite from BC, from 357.8 to 334.5 °C for BC-MZA3. Other aspects, i.e., water content, porosity, hemocompatibility and histology studies, also showed that the composite could potentially be used as a wound dressing.

## 1. Introduction

Burned skin wound healing is a complex process that requires the involvement of tissues, cell types and matrix components and time to heal [1]. Damaged skin areas are known to have a high risk of attracting infectious bacteria, which affect the natural wound healing mechanism [2,3]. Therefore, wound care with a wound dressing is essential for an effective healing process [4,5].

In order to accelerate the healing process, an active wound dressing was proposed in wound management because the wound dressing that was commercially available, e.g., gauze, does not play an active role in the wound healing process. Gauze was observed to potentially produce additional trauma when it was released from the wound bed due to its dried material properties. It also did not contain drugs that could help in reducing the time for wound closure. Meanwhile, an active wound dressing is known to be able to meet several requirements of wound management, such as protecting wounds from infections during the healing process [6,7,8] as well as creating a supportive environment for the regeneration of the epidermis for injured skin tissue by providing a barrier to prevent wound infection, binding and controlling the release of drugs, absorbing the fluid produced from the wound and reducing pain [8,9]. Previous studies have shown that films, hydrogels and foams formulated from different biopolymers can be used to accomplish these functions [4,10].

Bacterial cellulose (BC), a cellulose that is produced by *Acetobacter xylinum* strain bacteria, is a natural polymer that can be used in various biomedical applications. In addition to its purity, BC also has other superior properties compared to plant-based cellulose. Bacterial cellulose has high crystallinity due to its well-woven fiber structure, a high water-binding capability and biocompatibility [11,12,13]. Its high water-binding capability affects drug release controlling properties. This was reported through previous research and it has become an advantage to apply BC as the base material in biomedical applications, e.g., tissue engineering, blood vessel replacement, skin cell scaffolding and wound dressing, due to its drug release controlling properties [8,14,15,16,17]. The indication that drugs or active components can be trapped in BC films and released gradually was made apparent by the high number of wrinkles that appeared after the drying process. Thus, dried BC can be used as a film matrix for the slow release of active components [18,19].

*Zanthoxylum acanthopodium* fruit is known in Indonesia as andaliman. It is spicy in flavor and commonly used to eliminate the smell of raw fish and meat. *Z. acanthopodium* is a wild plant that grows in Tapanuli, North Sumatra, Indonesia, located at an altitude of 1500 m above sea level with a temperature between 15 and 18 °C [20]. The shape of the fruit resembles a green pepper, but its green color turns black when it dries. Previous studies confirmed that *Z. acanthopodium* fruit has anti-inflammatory, antioxidant and antimicrobial abilities [21,22]. They also documented the antibacterial activity of *Z. acanthopodium* fruit extracts against *B. stearothermophilus*, *P. aeruginosa*, *V. cholera*, *E. coli*, *S. aureus* and *Bacillus* [20,23].

Therefore, it is necessary to conduct research to make BC-based wound dressings loaded with micro-colloidal *Z. acanthopodium (MZA)* that can accelerate the wound healing process through the improvement of BC fiber pores, antibacterial activity and bioactivity by providing drugs for anti-inflammation. The formed composite was characterized and its potential as a wound dressing was studied using scanning electron microscopy (SEM), Fourier-transform infrared spectroscopy (FTIR), X-ray diffraction (XRD), thermogravimetric analysis (TGA), moisture content tests, porosity tests, hemocompatibility assessments and in-vivo tests.

## 2. Materials and Methods 

### 2.1. Materials

*Acetobacter xylinum,* the bacterial strain used in this research, was collected from the Polymer and Material Lab, Chemistry Postgraduate, Universitas Sumatera Utara, Medan, Indonesia. Coconut water and *Z. acanthopodium* fruit were purchased from a local market in Medan, Indonesia, while chemicals such as glucose, urea, NaOH and glacial acetic acid were purchased from Merck (Damstadt, Germany).

### 2.2. Method

#### 2.2.1. Production of MZA 

*Z. acanthopodium* fruit was collected, sorted, washed and drained. Next, *Z. acanthopodium* fruit was placed on paper and dried in an open space with no direct sunlight for around 10 days. MZA was produced by crushing the dried *Z. acanthopodium* fruit using a high energy milling device (HEM) in LIPI Serpong, Jakarta, Indonesia.

#### 2.2.2. In-Situ Fabrication of BC-Loaded MZA 

Glucose (10 g/L) and urea (5 g/L) were supplemented to the coconut water as a source of carbon and nitrogen, while glacial acetic acid (1 mL/L) was added later to adjust the medium pH to 5. Then, 5–25 g/L MZA was added into each flask to fabricate BC-MZA via an in-situ method. The medium and MZA mixture was stirred for 20 min with a mechanical stirrer to ensure that it was well combined. Then, it was sterilized in an autoclave. Finally, *Acetobacter xylinum* (0.1 v/v) was added to the medium and it was inoculated for 8 days.

#### 2.2.3. BC-MZA Composite Purification

The BC-MZA composite that floated on the top layer of the medium was harvested. Then, it was washed with distilled water and immersed in 2.5% NaOH for 24 h, according to the purification process performed by Gea et al. [24]. After 24 h, the BC-MZA composite was neutralized using aquadest and dried in an oven at 40 °C. Each sample was labeled as shown in Table 1. 

#### 2.2.4. Fourier-Transform Infrared Spectroscopy (FTIR)

Fourier-transform infrared spectroscopy (FTIR) characterization was done to determine the biopolymer’s functional group. Samples were characterized using Bruker Platinum-ATR, Alpha in wavelengths with a range of 4000 to 400 cm^−1^.

#### 2.2.5. X-Ray Diffraction (XRD)

The X-ray diffraction (XRD) of the sample was studied by scanning a 2θ angle sample in a 7–70° range, using a Shimadzu XRD-6100 diffractometer. For further investigation, Equation (1) was used to calculate the crystallinity index (CrI) for all samples.

(1) $$\mathrm{Cr}.I\;\left(\%\right)=\frac{\mathrm{Area}\;\mathrm{of}\;\mathrm{the}\;\mathrm{crystalline}}{\mathrm{Area}\;\mathrm{of}\;\mathrm{total}\;\mathrm{domain}}\cdot \;100\%$$

#### 2.2.6. Scanning Electron Microscopy (SEM)

The BC-MZA composites were characterized by using scanning electron microscopy (SEM) EDX EVO MA Zeiss Bruker operated at 20 kV. The analysis was conducted by characterizing the surfaces and layers in order to study the morphology of the composite that was formed.

#### 2.2.7. Thermogravimetric Analysis (TGA)

Thermogravimetric analysis (TGA) and derivative thermogravimetric analysis (DTGA) were carried out to characterize the thermal properties of the developed biopolymers. The thermal breakdown of the samples was analyzed by using EXTAR 7300 series, with a temperature range of 30–600 °C, under nitrogen gas conditions with a temperature rise of 10 °C/min.

#### 2.2.8. Moisture Content (Mc)

The moisture content of all samples was characterized by measuring the pure sample weight and the freeze-dried sample weight. The ability of the samples to bind water was calculated using Equation (2).

(2) $$\mathrm{Mc}\;\left(\%\right)=\frac{\mathrm{Weight}\;\mathrm{wet}\;\mathrm{samples}-\mathrm{Weight}\;\mathrm{dry}\;\mathrm{sample}}{\mathrm{Weight}\;\mathrm{wet}\;\mathrm{samples}}\cdot 100\%$$

#### 2.2.9. Porosity Assessment

The porosity of the sample was studied by calculating the data using Equation (3). Data were obtained by immersing the sample in 96% ethanol for 10 min.

(3) $$\mathrm{Porosity}\;\left(\%\right)=\frac{\mathrm{Initial}\;\mathrm{volume}\;\mathrm{of}\;\mathrm{ethanol}-\mathrm{Volume}\;\mathrm{of}\;\mathrm{ethanol}\;\mathrm{after}\;\mathrm{sample}\;\mathrm{was}\;\mathrm{taken}}{\mathrm{Volume}\;\mathrm{of}\;\mathrm{ethanol}\;\mathrm{when}\;\mathrm{sample}\;\mathrm{was}\;\mathrm{immersed}-\;\mathrm{Volume}\;\mathrm{of}\;\mathrm{ethanol}\;\mathrm{after}\;\mathrm{sample}\;\mathrm{was}\;\mathrm{taken}}\cdot 100\%$$

#### 2.2.10. Hemocompatibility Test

The hemocompatibility test was done by washing the anticoagulant in horse blood with 0.9% saline solution by centrifugation at 3000 rpm for 10 min. Then, the disk-shaped sample with an 8 mm diameter was immersed in a horse blood cell solution at 37 °C for 2 h by an agitation process. Distilled water was used as a negative control while normal saline solution was used as a positive control. A spectrophotometer was used to measure the absorbance of the samples at 540 nm. Finally, the percentage of hemocompatibility was calculated by Equation (4).

(4) $$\mathrm{Hemocompatibility}\;\left(\%\right)=\frac{\;\mathrm{Samples}\;\mathrm{absorbance}-\mathrm{Negative}\;\mathrm{control}\;\mathrm{absorbance}}{\mathrm{Positive}\;\mathrm{control}\;\mathrm{absorbance}-\;\mathrm{Negative}\;\mathrm{control}\;\mathrm{absorbance}}\cdot 100\%$$

#### 2.2.11. In-Vivo Test (Full-Thickness Burn Wound)

In order to test the efficacy of BC-MZA composites in accelerating the burn healing process, an in-vivo test was performed on 28 male and female rats (*Rattus norvegicus*) aged 3 months, with body weights of 150–180 g. The animal testing procedure performed in this study was approved by the Animal Research Ethics Committees (AREC) No.0731/KEPH-FMIPA 2019 of Universitas Sumatera Utara, Indonesia. All research processes were conducted in the animal research lab at Universitas Sumatera Utara, following the university guidelines. The full-thickness burn wound was prepared first by shaving the skin at the back area, where treatment would be given later. After this, the rats were anesthetized using xylazine (5.0 mg/kg) and ketamine (35.0 mg/kg). Next, the skin area was cleaned with chlorhexidine, povidone iodine and alcohol (70%). The full-thickness burn wound was made on the cleaned area by placing a 20 × 20 mm rectangular piece of hot metal perpendicular (80 °C) to the rat dorsum within one second, precisely at the position between the last thoracic vertebra and the first sacrum. The rats were randomly divided into seven groups of 4, as follows: control (-), BC, BC-MZA1, BC-MZA2, BC-MZA3, BC-MZA4 and BC-MZA5. The control (-) group did not receive any treatment. In this test, BC and BC-MZA samples were used without dried treatment in order to minimize pain and stimulate the healing process.

#### 2.2.12. Wound Closure Assessment

Burns were observed every three days, from day 0 to day 21. The most significant changes in the area of the wound were observed on days 7, 14 and 18. The wounds were measured using a caliper and recorded by a camera. After this, the percentage of wound closure was calculated using Equation (5).

(5) $$\mathrm{Wound}\;\mathrm{Reduction}\;\left(\%\right)=\frac{\mathrm{Wound}\;\mathrm{Area}\;\mathrm{Day}\;0-\mathrm{Wound}\;\mathrm{Area}\;\mathrm{Relative}\;\mathrm{Day}}{\mathrm{Wound}\;\mathrm{Area}\;\mathrm{Day}\;0\;}\cdot 100\%$$

#### 2.2.13. Microbiological Examination

The burned skin area from the group of rats treated with bandaged (positive control), bioplacento, BC-MZA3 and the group with no treatment (negative control) were swabbed on days 4, 8, 12 and 16 for bacterial analysis in the microbiology lab. Each sample was dissolved in 2 mL of normal saline and subsequently vortexed in a 10-fold serial dilution. A volume of 1000 μL dilution of the sample was spread and flattened on a tryptic soy agar (TSA) surface. The culture was left for 24 h in an incubator at 37 °C, before the number of bacterial colonies were finally counted. 

#### 2.2.14. Histopathological Observation

After 21 days, all tissue areas treated were collected for histopathological observation. The samples were set in 10% buffered formalin, prepared and coated in paraffin. The visualization of the paraffin-embedded specimens was observed under microscopic light after cutting and marking with Masson’s trichrome (MT) stains.

## 3. Results

### 3.1. FTIR BC-MZA Composites

**Figure 1:**
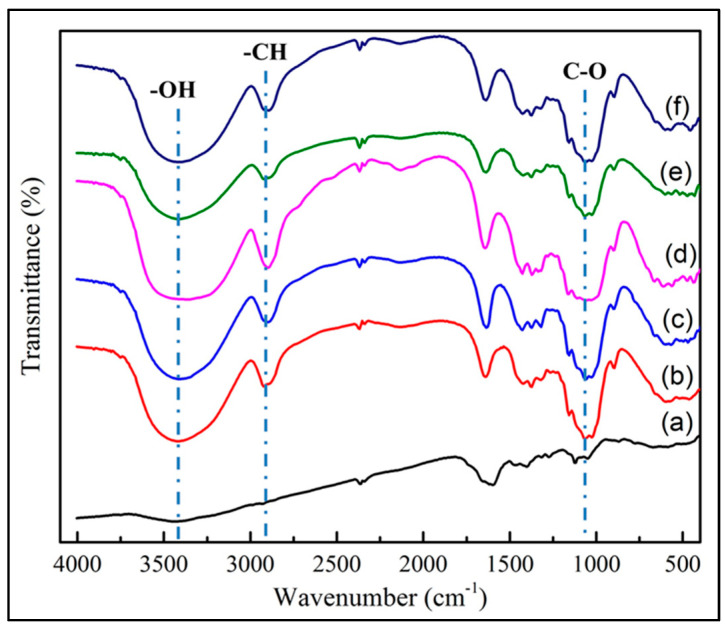
FTIR spectra of (a) BC, (b) BC-MZA1, (c) BC-MZA2, (d) BC-MZA3, (e) BC-MZA4 and (f) BC-MZA5.

The FTIR test results indicated in Figure 1 show that the three typical bands commonly found in pure BC were the band at 3200-3700 cm^−1^, which indicated the presence of OH groups, the band at 2900–2950 cm^−1^, which showed C-H bonds, and the band at 1050 cm^−1^, which assigned C-O bonds [25,26]. These three bands were also observed in the BC-MZA composite spectra, as shown in Figure 1b–f, but in slightly different peak intensities. In the BC-MZA composites, the intensity of the OH band was observed to be higher. This is due to the presence of flavonoid groups that provide antioxidant properties in Z. acanthopodium, which, in previous research, had been reported to be able to prevent inflammatory reactions [20,21].

### 3.2. XRD of BC-MZA Composites

**Figure 2:**
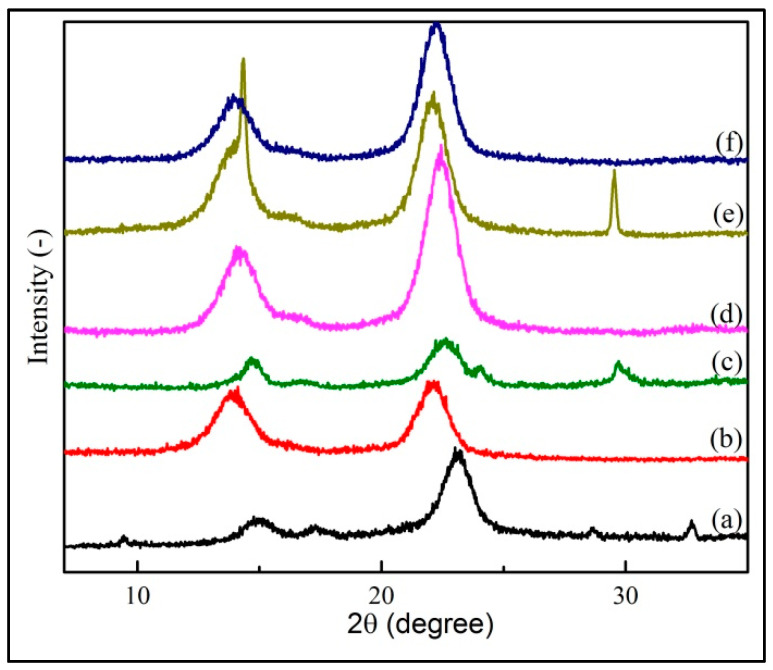
X-Ray diffraction patterns of (a) BC, (b) BC-MZA1, (c) BC-MZA2, (d) BC-MZA3, (e) BC-MZA4 and (f) BC-MZA5.

The XRD test results displayed in Figure 2 indicated that the addition of MZA in BC via the in-situ technique still maintained the presence of the BC’s typical peaks in the composite, which were the peaks at 14° and 22° [27], but they were observed at lower intensities. As a result, the composite possessed a lower crystanility index (CrI). From the calculated XRD crystallographic plane, it can be analyzed that the addition of 5 g/MZA in the BC-MZA1 sample possessed a CrI value of 73.30%, which indicated that it had a lower peak intensity compared to the peak in the pure BC with a 82.88% CrI value. The lowest peak intensity was seen in the addition of 10 g/L MZA with a CrI value of 70.27%. The data are shown in Table 2. A decreasing trend in the CrI values of the composites was caused by the existence of MZA trapped in the BC fibers, which is known to affect the arrangement of the fibers in crystallization process. This is consistent with previous research which mentioned that the presence of fillers in BC fibers would decrease the CrI value of the composite formed [28,29]. 

### 3.3. Morphology of BC-MZA Composite

**Figure 3:**
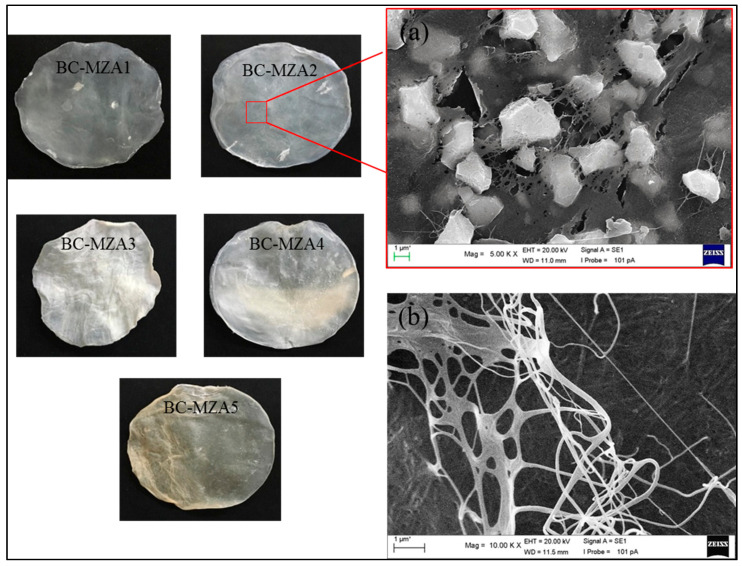
Morphological photo and SEM images of (**a**) BC-MZA2 composite and (**b**) BC.

The addition of active components into the BC fibers resulted in wrinkle formation in the BC-based composites (Figure 3). This result is consistent with previous research, which showed that BC can be used as a matrix film where trapped drugs or active components were characterized by observing the creation of wrinkles after the drying process. This suggests that the BC matrix can handle the gradual release of drugs or active components [18,19].

A nanofiber structure can be seen from the SEM analysis imaging of neat BC, as shown in Figure 3b. The addition of MZA into the medium showed that MZA was well-trapped in the BC fibers (Figure 3a). The presence of MZA in the BC was observed to have interrupted the fiber structure. This happened because MZA was loaded into the BC fibers via in-situ methods and it disrupted the well-woven BC synthesized by *A. xylinum*. These images correlated with the XRD data, which displayed a lower CrI percentage in the composites than in the neat BC. The results indicated that the in-situ fabrication of BC-MZA composites can be carried out with good penetration of MZA into the BC fibers.

### 3.4. TGA/DTGA BC-MZA Composites

**Figure 4:**
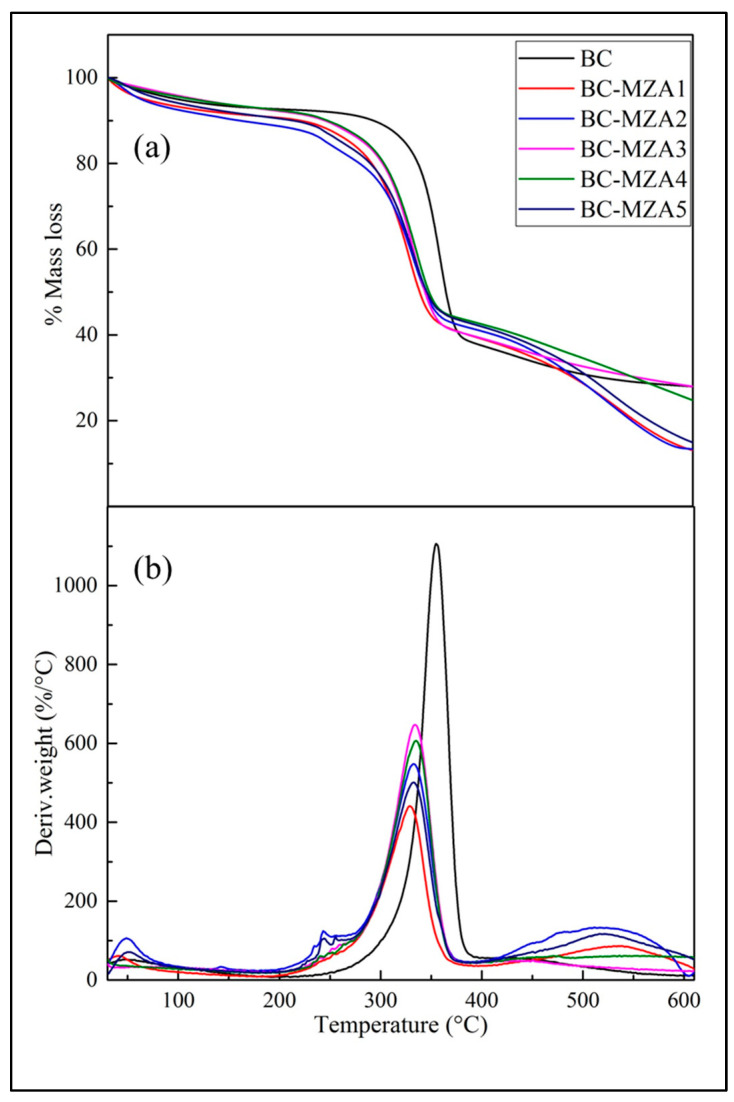
(**a**) TGA and (**b**) DTGA curves of BC, BC-MZA1, BC-MZA2, BC-MZA3, BC-MZA4 and BC-MZA5.

Based on the TGA and DTGA curves in Figure 4, the thermal degradation of BC occurred in three stages. The first stage occurred at 30–110 °C, which could be assumed as the evaporation process of the water contained in the BC. The second stage occurred at 220–390 °C, at which BC experienced a major loss of mass during the thermal decomposition process. Then, the third stage occurred at the 435–600 °C temperature range, which was known as the carbonization process [11]. From the curves, these three stages of thermal breakdown were also experienced by the BC-MZA composites. However, there were significant differences observed in the second stage. This stage took place at the temperature range of 180–365 °C for the five composite variations. The remaining residues were recorded at the end of the process for BC, BC-MZA1, BC-MZA2, BC-MZA3, BC-MZA4 and BC-MZA5, with the respective results as follows: 27.98%, 12.96%, 13.32, 27.93%, 24.49% and 14.63% (Table 3). 

The DTGA curve shows that the maximum temperature in the thermal degradation stage with the major loss of mass in BC occured at 357.8 °C. Meanwhile, the peaks of the BC-MZA composite were observed at 328.7, 332.5, 334.5 and 334.4 and 332.2 °C for BC-MZA1, BC-MZA2, BC-MZA3, BC-MZA4 and BC-MZA5, respectively, as listed in Table 3. This showed that the presence of MZA in the BC fibers caused the composite to possess lower T_max_. This is consistent with the TGA curve, in which the second stage experienced by the BC-MZA composite was at 150–350 °C. This result may be correlated with the presence of MZA in the fibers, which made thermal breakdown more easily experienced by the natural product. In addition, the T_max_ shift to lower temperatures was also attributed to the lower CrI percentage of the composite compared to the neat BC, since CrI is one of the structural parameters that affects thermal degradation [30].

### 3.5. Moisture Content (%) and Porosity (%)

The results of the moisture content evaluation showed that the addition of MZA into the BC fibers did not significantly affect the reduction in moisture content (Table 3). The moisture content values for each sample were 98.9 ± 0.15, 97.8 ± 0.08, 97.5 ± 0.10, 96.7 ± 0.07, 96.5 ± 0.08 and 95.2 ± 0.05 for BC, BC-MZA1, BC-MZA2, BC-MZA3, BC-MZA4 and BC-MZA5, respectively. The results were advantageous for the use of composites as wound dressings, because the moisture level affects the adhesiveness of the wound dressing to the wound bed. The risk of injury can increase during the removal of wound dressing in cases where it is too dry [7].

The addition of nanoherbal andaliman to BC was not known to significantly influence the decrease in the porosity of the composites. The porosity values of the samples are presented in Table 3, and the values for BC, BC-MZA1, BC-MZA2, BC-MZA3, BC-MZA4 and BC-MZA5 were 78.2 ± 0.13, 77.8 ± 0.08, 77.5 ± 0.02, 76.3 ± 0.05, 74.2 ± 0.06 and 73.9 ± 0.08, respectively.

### 3.6. Hemocompatibility (%)

The hemocompatibility test was conducted to investigate the percentage release of hemoglobin into the plasma due to the damages in erythrocytes and analyze the biocompatibility properties of the composites. Evaluation of six samples (Table 4 showed that there were five non-hemolytic samples, with hemocompatibility (%) values between 0–2% for BC (1.52% ± 0.02%), BC-MZA1 (1.52% ± 0.01%), BC-MZA2 (1.55% ± 0.03%), BC-MZA3 (1.58% ± 0.03%) and BC-MZA4 (1.7% ± 0.02%). Then, the sample labelled as BC-MZA5 was considered to possess slightly hemolytic properties, with a hemolysis percentage of 2.05% ± 0.05%. The results referred to the American Society for Testing and Materials (ASTM F756-00, 2000) [31]. Therefore, the BC, BC-MZA1, BC-MZA2, BC-MZA3 and BC-MZA4 samples proceeded to the in-vivo tests discussed in the next section, while BC-MZA5 was not tested since it was classified as a slightly hemolytic material.

### 3.7. Microbiological Examination

**Figure 5:**
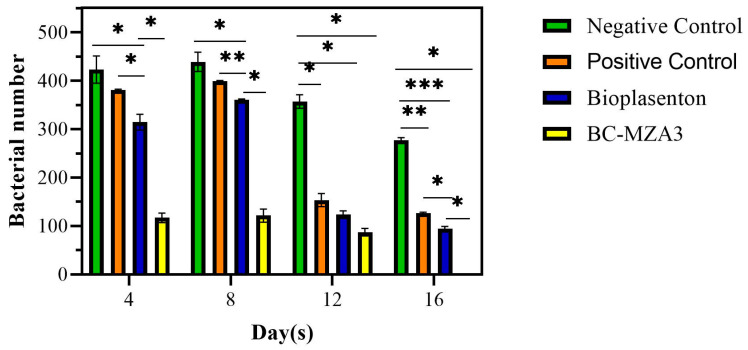
Number of bacteria on days 4, 8, 12 and 16 from different treatment groups (* *P* < 0.033, ** *P* < 0.002, *** *P* <0.0002; d).

A microbiological test was carried out on the wounds to confirm the presence of certain bacteria and the ability of the wound dressing to inhibit bacterial growth. The data in Figure 5 show that the composites were effective in slowing down bacterial growth in burns, in contrast to untreated wounds (negative control), bandaged wounds (positive control) and bioplacenton-treated wounds. On day 16, no bacteria were observed on the agar media from the treated groups using the BC-MZA3 composites, while bacteria were observed in the negative control group (277 ± 8), positive control group (126 ± 5) and bioplacenton-treated group (94 ± 7).

### 3.8. In-Vivo Test of BC-MZA Composites

#### Wound Closure

**Figure 6:**
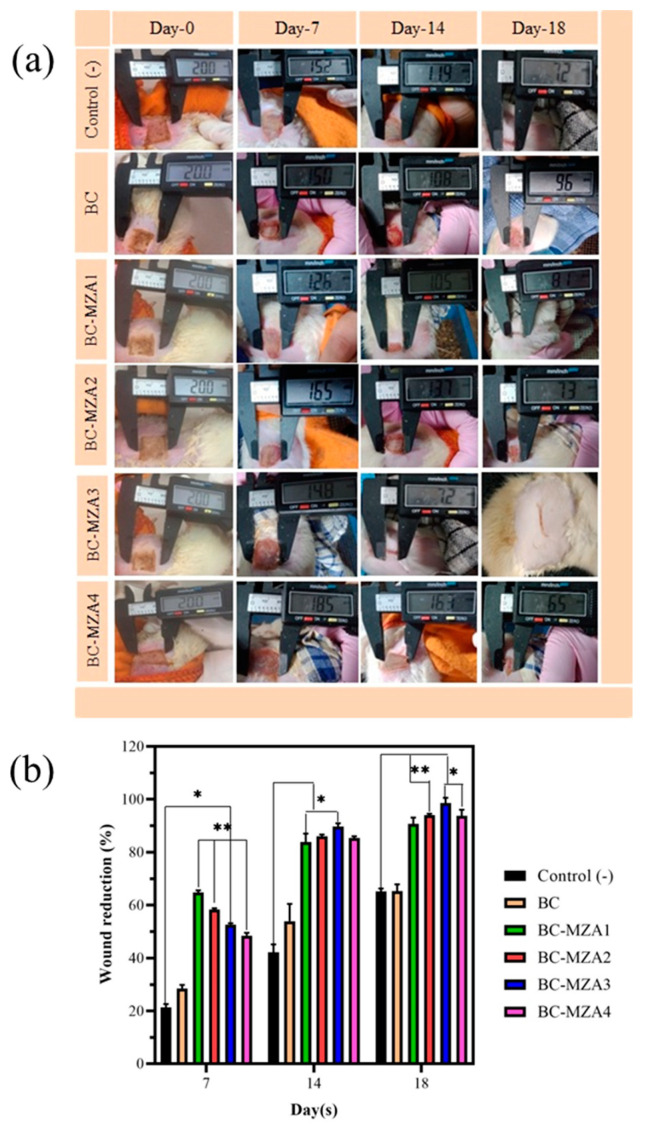
(**a**) Wound appearance and (**b**) wound reduction percentage (* *P* < 0.033, ** *P* < 0.002; d).

Rats were used to assess the efficacy of BC-MZA composites to treat burns. From the seven groups of rats that were tested, on day 7, each sample had a wound closure of 20.45% ± 2.22%, 29.45% ± 2.10%, 65.35% ± 1.10%, 58.65% ± 0.65%, 52.97% ± 0.7%, 47.63% ± 1.83% and 42.18% ± 3.05% for control (-), BC, BC-MZA1, BC-MZA2, BC-MZA3, BC-MZA4 and BC-MZA5 controls, respectively. The largest percentage of wound closure was observed in the group of rats treated with the BC-MZA1 composite. The observation was carried out until day 18, when it was found that the group of rats treated with BC-MZA3 had 97.70% ± 2.18% burn closures. Meanwhile, the remaining groups had experienced only 64.40% ± 1.50 %, 67.12% ± 2.70%, 89.02% ± 2.37%, 93.52% ± 0.80%, 92.03% ± 2.35%, and 90.15% ± 0.70% wound closure for control (-), BC-MZA1, BC-MZA2, BC-MZA4 and BC-MZA5 controls, respectively (Figure 6). The group which received BC-MZA1 treatment experienced the highest level of wound closure on day 7 but only had a wound closure percentage of 89.02% ± 2.37% on day 18, because there was a reduction in nanoherbal content. Therefore, the BC-MZA1 composite could not continue to provide drugs during the wound healing process. From the observations, it could also be seen that the group of rats treated with BC-MZA composites experienced a significant amount of healing compared to the group of rats treated with the control (-) and BC. This is in line with a number of studies which have suggested that a good wound dressing plays an active role in removing exudate wounds, preserving the moisture in the area, promoting air circulation to the wound, possessing antibacterial properties and providing drugs that can accelerate the wound healing process [4,5,10].

### 3.9. Histopathological Observation

**Figure 7:**
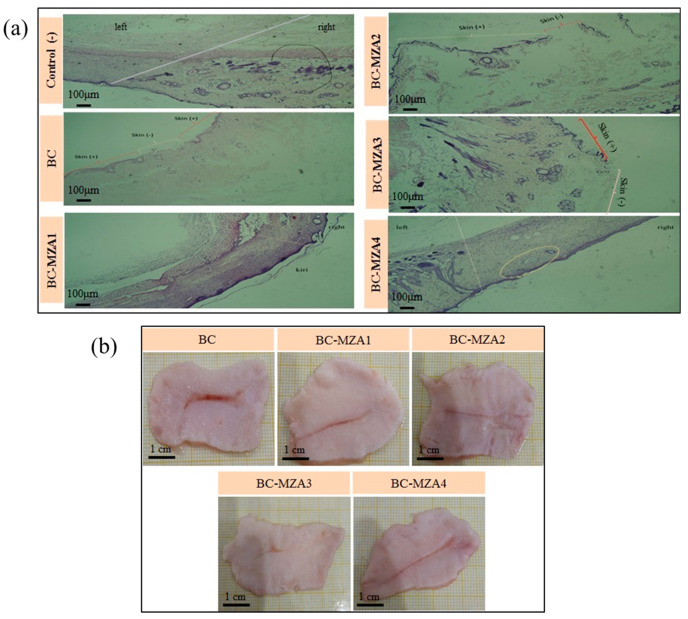
(**a**) Histopathological observation and (**b**) burned skin tissue granulation photos.

Histopathological observation (Figure 7a) was evaluated by analyzing the re-epithelialization, fibroblast immigration, inflammatory cell infiltration and connective tissue synthesis of the regenerated tissues on the 18th day of burned skin. Healed burn skin tissue samples treated with the control (-) were observed to have grown epidermis (stratified squamous epithelium), fibroblast cells and hair follicles (-). Meanwhile, the tissues treated with BC exhibited discontinuous epithelial layers with connective tissues, hair follicles and sebaceous glands (+). Moreover, wounds treated with BC-MZA1 were seen to have denser keratin, epidermis (stratified squamous epithelium) and fibroblast cells. This was due to the presence of antioxidants in MZA, which suppressed inflammatory cell infiltration and accelerated the wound healing process [32]. In this sample, hair follicles and blood vessels were found. This also indicated that MZA played an important role in the wound healing process since it could support vascularization. It is already known that vascularization can promote fibroblasts, bring nutrition and supply macrophages and other monocytes which can manage low level inflammation with anti-bacterial properties [33]. Compared to BC-MZA1 treatment, skin tissues treated with the BC-MZA2 composite were observed to have no epithelial layer, hair follicles or sebaceous glands, whereas skin tissues treated with the BC-MZA4 composite were observed to be almost without keratin, epithelial layers (+) along the tissues, connective tissues consisting of collagen or fibroblast masses. Meanwhile, burned skin tissues treated with the BC-MZA3 composite displayed the best wound healing process among the samples. This was indicated by the complete formatting of epithelial layers with connective tissues which were observed to mimic normal skin. This histopathological observation was also supported by the granulation tissues of the burned skin in rats, as shown in the photographs, which were harvested after 18 days. From the pictures, skin tissues treated with BC-MZA3 (Figure 7b) appeared to have softer scars than the other skin tissues treated by other samples. In other words, based on our in-vivo observations, the granulation tissue of BC-MZA3 showed the best wound closure performance among all samples.

## 4. Conclusions

In conclusion, BC-MZA composites were successfully fabricated via an in-situ method. This is supported by the characterization results obtained from FTIR, XRD, TGA/DTGA and SEM. BC-MZA composites were also found to be potential materials for wound dressings, after moisture content, porosity, hemocompatibility, microbiological assessment and in-vivo testing were conducted. In this study, in-vivo testing showed that the composite with the 15 g/L MZA composition (BC-MZA3) was the optimal composite to be applied as a wound dressing. Based on the histopathological observations, the burned tissues treated with the BC-MZA3 composite displayed a complete formatted epithelial layer and had softer scars in the granulation tissues. This study also found that the addition of 25 g/L MZA (BC-MZA5) could not be recommended as a wound dressing, since the material produced was classified as slightly hemolytic.

## Figures and Tables

**Table 1:** Name and treatment variation for each sample.

Sample Name	BC (g/L MZA)
BC-MZA1	5
BC-MZA2	10
BC-MZA3	15
BC-MZA4	20
BC-MZA5	25

**Table 2:** Crystallinity index (CrI) data of BC, BC-MZA1, BC-MZA2, BC-MZA3, BC-MZA4 and BC-MZA5.

Sample	CrI (%)
**BC**	82.88
**BC-MZA1**	73.30
**BC-MZA2**	70.27
**BC-MZA3**	75.84
**BC-MZA4**	75.22
**BC-MZA5**	72.85

**Table 3:** Onset temperature (°C) and residual mass (%) data of BC, BC-MZA1, BC-MZA2, BC-MZA3, BC-MZA4 and BC-MZA5.

Sample	BC	BC-MZA1	BC-MZA2	BC-MZA3	BC-MZA4	BC-MZA5
**T_max_ (°C)**	357.8	328.7	332.5	334.5	334.4	332.2
**Residual mass (%)**	27.98	13.08	13.32	27.93	24.49	14.63

**Table 4:** Moisture content (%), porosity (%) and hemocompatibility (%) data of BC, BC-MZA1, BC-MZA2, BC-MZA3, BC-MZA4 and BC-MZA5 (n = 4).

Sample	Moisture Content (%)	Porosity (%)	Hemocompatibility (%)
**BC**	98.9 ± 0.15	78.2 ± 0.13	1.52 ± 0.02
**BC-MZA1**	97.8 ± 0.08	77.8 ± 0.08	1.52 ± 0.01
**BC-MZA2**	97.5 ± 0.10	77.5 ± 0.02	1.55 ± 0.03
**BC-MZA3**	96.7 ± 0.07	76.3 ± 0.05	1.58 ± 0.03
**BC-MZA4**	96.5 ± 0.08	74.2 ± 0.06	1.7 ± 0.02
**BC-MZA5**	95.2 ± 0.05	73.9 ± 0.08	2.05 ± 0.05
